# It Takes A Village; Involvement of Village Health Teams to Develop Tools and Resources to Communicate about Antiretroviral Use in Pregnancy and Breastfeeding at Community Level in Uganda.

**DOI:** 10.12688/wellcomeopenres.19088.1

**Published:** 2023-11-09

**Authors:** Esther Alice Nalugga, Mercy Kukundakwe, Robinah Tibakanya, Elizabeth Tindyebwa, William Baluku, Kenneth Mulindwa, Simon Peter Asiimwe, Adelline Twimukye, Catriona Waitt

**Affiliations:** 1 Infectious Diseases Institute, Makerere University College of Health Sciences, Kampala, Uganda; 2 Hoima District Forum of PLHIV Networks, Hoima, Uganda; 3 Department of Pharmacology and Therapeutics, University of Liverpool, Liverpool, UK

**Keywords:** Public involvement, Village health teams, Breastfeeding, Co-creation, Job-aids.

## Abstract

**Background:**

Research findings must be communicated to the populations who will benefit from them, in a manner that is accessible and understandable.

**Aims:**

We recently generated novel data on medication use in breastfeeding. A Faculty of Health and Life Sciences (Liverpool) grant enabled work with a team of Village Health Teams (VHTs) in Hoima, rural Uganda, to co-create related communication materials for use in their house-to-house visitation and health education.

**Methods:**

After an initial workshop from 24th to 26th March 2021, training and review of draft materials, 10 VHT pairs visited 50 households in Hoima district. Basic demographic data were collected alongside preferred communication methods. VHTs provided feedback and re-design of materials commenced. This included dramatization of scenarios and photography. A second round of house-to-house visitation informed final adjustments. We conducted focused group discussions and a dissemination workshop attended by VHTs, local healthcare leaders and journalists was hosted on 16th – 17th June 2022.

**Results:**

Most households (74%) had a breastfeeding baby. Majority could read and had access to radios (60%), but not to smartphones (58%) or television (86%). Most preferred verbal and visual aids for health education, and requested images of “people who look like us”.

Final co-created materials included posters in English and Runyoro and laminated ‘job aids’ in both languages . These continue to be in active use. VHTs and community members requested future projects of this nature.

**Conclusion:**

Healthcare communication to communities must be accessible and clear. Representation of images that the public can identify with is important. Co-creation workshops were successful in rural Uganda, and pave the way for future collaborative, participatory research.

## Introduction

It is ethically essential that research findings are communicated to the communities who will benefit from the results, in a manner that is clear and accessible to them. However, researchers often focus their energy on research dissemination within the scientific community ^1^. The measurable outputs of conference presentations and peer-reviewed publications are valued by funders and academic institutions, but are rarely of direct relevance to the population where the research has been undertaken ^2^. This has the potential to perpetuate any imbalance in power dynamics and reduce the desired impact of the research itself. Public engagement and involvement is increasingly emphasised in order to build mutually beneficial dialogue and establish partnerships of trust ^3^. Co-production is an approach to working together in equal partnership and for equal benefit. This brings together different forms of lived or living and learnt (personal and professional) knowledge, understanding, and experience, for better outcomes and mutual benefit ^4^.

Since the 1978 Alma-Ata declaration, which reaffirmed access to health as a fundamental human right and identified primary healthcare as the key to the attainment of the goal of health for all, global partners have focused on the role of Community Health Workers in achieving this objective ^5^. In Uganda, the 1999 national health policy included community empowerment and mobilisation for health as a key element, building a strategy based on Village Health Teams (VHTs) ^6^. These draw on the ‘natural helper model’, based on the premise that within every community, an informal helping network already exists ^7^. Through selection of individuals who are already trusted within communities, further basic training can ensure that a trained ‘natural helper’ can act as a representative of each network within a community ^7, 8^. Some of the strategies recommended by the World Health Organisation (WHO) include home visits conducted by VHTs as a means to communicate relevant health messages necessary to improve maternal and child health. VHTs often form a bridge between healthcare workers and community and are more trusted by rural communities ^9^. This concept of peer education from a trusted community member may be of particular importance for young mothers who may struggle to access the information they require, particularly in settings of low literacy and hierarchical, patriarchal social structures which tend to disempower women.

Worldwide, it is estimated that around half of all women require some form of medication during breastfeeding ^10^. In Uganda, the HIV prevalence in women of childbearing potential was 7.1% in 2020-2021, with the highest rates of infection among adolescent girls and young women ^11^. The lowered immunity that occurs during pregnancy leaves women susceptible to other infections such as tuberculosis ^12^ and malaria ^13^. However, despite the frequent need for lifesaving medication during breastfeeding, many medications lack data on the transfer of drug through breastmilk to the infant. This lack of firm data can cause anxiety about the safety of taking medication during breastfeeding, and women may risk untreated disease rather than expose their infant to a potential risk ^14^. In response to this lack of data, studies which SPA, WB, AT and CW have been involved in, including the Wellcome Clinical Research Career Development fellowship MILK (Maternal and Infant Lactation Pharmacokinetics) ^15, 16^ and the DolPHIN1 (NCT02245022) ^17, 18^ and DolPHIN-2 (NCT03249181) ^19^ clinical trials have provided data from Ugandan and South African populations demonstrating that many antiretroviral drugs taken in pregnancy and breastfeeding are safe but that small amounts of drug can be measured in the breast milk and in the infant. Initial public engagement work was undertaken during World Breastfeeding Week 2019, including a national celebration in Kiboga district, rural Uganda, attended by a delegation from the Ugandan Ministry of Health. Informal feedback from that event highlighted the need to communicate about our work at community level including rural districts.

Drawing all of this together, co-production with a network of VHTs in rural Uganda seemed the ideal approach to genuinely engage the target population with findings arising from our research. This project aimed to use the local expertise and networks of VHTs to determine priorities and concerns regarding breastfeeding among women living with HIV at community level in order to shape future research. Furthermore, we sought to co-create a suite of resources to communicate about the priority health topics, including medication use during breastfeeding.

## Methods

### Ethical approval and consent

This project was defined as a community engagement pre-research exercise as per Uganda National Council of Science & Technology (UNCST) guidelines, which includes but is not limited to ‘community mapping, identifying and consulting with the relevant key stakeholders, meeting with local/community leaders, identifying the values and norms of the community, introduction of the research team to the community, mobilization of the target audience, community sensitization, and formative consultations where applicable’. Ethical approval was not sought for this engagement phase of the project as it was not considered ‘research involving humans as research participants’, based on UNCST definitions, but rather focused on sharing key messages, with the communities, from previously conducted research studies.

Verbal, informed consent instead of written consent was acquired as we built upon the standard processes used by VHTs in conducting home visits in these communities. We worked within their already existing structures as this would ensure continuity and sustainability of the activities after the project had ended. Written, informed consent was acquired for the publication of identifiable images.

### Selection of Hoima district

Hoima district is a predominantly rural district located in western Uganda, 230 kilometres from Kampala, Uganda’s capital city. In 2020, Hoima district had an estimated population of 374,500 people among whom 24% are females of reproductive age (15–49 years). According to the Uganda AIDS Commission, the prevalence of HIV in Hoima was 6.6%, the second highest in the western region and slightly lower than that of Kampala ^20^. Similarly, the Uganda Population-based HIV Impact Assessment (UPHIA) conducted in 2020–2021 reported that HIV was more prevalent among women 15 years and older and thus this made the population in Hoima suitable ^11^. Furthermore, existing relationships with the Hoima district coordinator of people living with HIV/AIDS Networks indicated feasibility. Hoima has a strong network of VHTs who have basic familiarity with research principles ^21^.

### Selection of VHTs

The project used the VHTs approach who comprised of lay, community members who had previously undergone short, focused trainings to empower them support health related programs (for example supporting postnatal mothers in accessing healthcare, immunization campaigns and health education among other activities). 

A total of 20 Village Health Team (VHTs), attached to various health facilities in Hoima District, Uganda, were selected in consideration of their enthusiasm in their respective communities and health facilities.

### Structure of co-creation activities

Basic materials were already in existence following the 2019 World Breastfeeding Week events. These included several posters and flyers communicating basic information about antiretroviral medication and breastfeeding, available in both English and Luganda. These were used as a proposed starting point from the evaluation.

**Figure 1.**
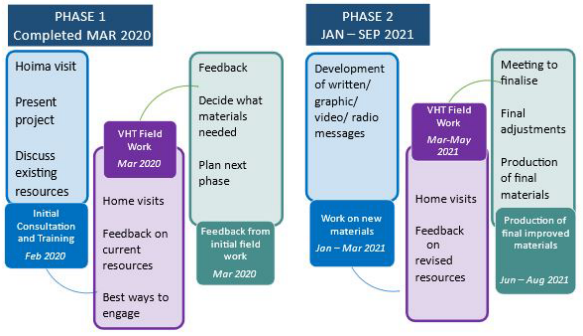
Study Design; Two cycles, starting with evaluation of existing materials from WBW2019, followed by refinement then evaluation of tailored resources for the community. Dates updated following COVID-19 disruptions.

The project was designed to be conducted in two phases, allowing a reflective, iterative approach to co-creation, as shown in Figure 1. At all stages we sought to remain flexible and adaptable to the emerging priorities and direction indicated by the VHTs.

An initial workshop enabled establishment of relationships and baseline knowledge with regard to the management of HIV during pregnancy and breastfeeding, early infant nutrition and public health measures to promote health. Following this, the VHTs formed into pairs and embarked upon house-to-house visitation using the resources from WBW2019. Basic demographic information including educational attainment, literacy and access to technologies was collected together with informal feedback on the content of the existing materials. In addition, the VHTs made notes about the key topics about which the community members requested further information.

Feedback was then provided to the team from IDI. From this, priority topics and preferred means of communication had been established. Improved materials were developed and brought to a subsequent workshop at which additional training was provided to the VHTs at their request. A second round of house-to-house visitation with near final versions of the resources took place and minor feedback was provided to the IDI team by email.

Upon completion, translation and printing of the final materials, a dissemination workshop was held in Hoima district, attended by the VHTs, the District Health Officer and the District Education Officer together with local journalists. Final training on the use of the co-created materials was provided.

## Results

### Phase 1

An initial visit was made to Hoima district where we met with the pre-selected VHTs (6 males, 7 females) and introduced the project goals and conducted the VHTs training. During this visit, we also assessed the VHTs’ knowledge on breastfeeding in the context of HIV. The VHTs were then dispatched into the community in pairs to conduct door to door baseline assessment of the community needs.

***Demographics.*** A total of fifty homes, with a median of five individuals in each household, were visited during the home visitation exercise. Among these, 94% were women and the median age was 28 years. Majority were married (48%) and 84% had Runyoro- Rutooro as their main primary language, as illustrated in Table 1.

**Table 1.** Baseline home visitation exercise.

Variable	N= 50
**Age in years, median (IQR)**	28 (16-57)
**Sex, n (%)** Male Female	3 (6) 47 (94)
**Marital status of person visited, n (%)** Single, no regular partner Single, long-term(>6months) partner Married Separated/divorced Widowed	8 (16) 14 (28) 24 (48) 3 (6) 1 (2)
**Primary languages used, n (%)** Luganda English Runyoro- Rutooro Others	3 (6) 2 (4) 42 (84) 3 (6)
**Primary bread winner, n (%)** Person visited Parent Spouse	11 (22) 25 (50) 14 (28)
**Number of dependents in the household, mean** ** (SD)**	4.82 (2.59)
**Number of adults (>18years), mean (SD)**	2.38 (1.28)
**Number of children, mean (SD)**	2.98 (1.78)
**Breastfeeding baby, n (%)** Yes No	37 (74) 13 (26)
**Level of understanding n (%)** Ability to read Ability to write Ability to reflect of oral communication Ability to reflect on visual communication	33 (66) 5 (10) 8 (16) 4 (8)
Others	
**Access to smart phones in the household, n (%)** Yes No	21 (42) 29 (58)
**Access to TV screens in the household, n (%)** Yes No	7 (14) 43 (86)
**Access to radios in the household, n (%)** Yes No	30 (60) 20 (40)
**Social places commonly attended by household** ** members, n (%)** Places of worship Clubs and groups Other	42 (84) 2 (4) 6 (12)

The households visited had an average of five dependents: two adults and three children, with majority (74%) having a breastfeeding baby. Majority could read, while majority had no access to smart phones (58%) and TV screens (86%) but had access to radios (60%). Places of worship were the commonly visited social places (84%).

Most of the homes visited preferred verbal and visual aids to help in health education. The health education topics for pregnant/ breastfeeding mothers and their partners similarly ranged from family planning to nutrition, breastfeeding, prevention of mother to child transmission (PMTCT) of HIV, stigma, infant and young child feeding practices (IYCF) and gender-based violence (GBV).

***VHTs’ knowledge assessment.*** At baseline 13 VHTs completed the knowledge questionnaire ^22^, median age 40 years (38–55), 53.8% female. Of these, majority, were confident in providing information about breastfeeding in HIV, although almost a quarter (23.1%) were uncertain. Interestingly, all the VHTs reported that it was fine for a baby born to an HIV positive mother to breastfeed and agreed that the father of the baby has a role to play in breastfeeding the baby, as illustrated in Table 2.

**Table 2.** Knowledge assessment of VHTs engaged in the project.

Variable	Baseline, N=13	Project end, N=16
**Age in years, median (IQR)**	40(38-55)	43(32-79)
**Sex , n (%)** Male Female	6(46.2) 7(53.8)	4(25) 12(75)
**Number of Years as VHT, n (%)** 2–5 5–10 More than 10	3(25) 3(25) 6(50)	4(25) 7(43.8) 5(31.2)
**Level of Education, n (%)** P5-P7 S1-S4 S6	1(7.7) 10(76.9) 2(15.4)	2(12.5) 11(68.8) 3(18.7)
**How confident are you in providing** ** information to a breastfeeding mother, n (%)?** Very confident Confident Uncertain Very uncertain	1(7.7) 8(61.5) 3(23.1) 1(7.7)	9(56.2) 7(43.8) 0(0) 0(0)
**Is it okay for a baby born to an HIV positive** ** mother to breastfeed, n (%)?** Yes	13(100)	16(100)
**Can a baby get HIV from breastfeeding, n (%)?** Yes No Not sure	10(76.9) 2(15.4) 1(7.7)	16(100) 0(0) 0(0)
**What is the recommended feeding option to** ** babies born to HIV positive mothers, n (%)** Only breast milk Both breast milk and other drinks Cow’s milk/formula	7(53.8) 5(38.5) 1(7.7)	16(100) 0(0) 0(0)
**What is the recommended time for initiation ** **of breastfeeding of a newborn baby, n (%)** Within 1 hour of birth After 1 day Any time	10(76.9) 1(7.7) 2(15.4)	15(93.8) 0(0) 1(6.2)
**Does the father of the baby have a role to play** ** in breastfeeding the baby, n (%)?** Yes No	13(100) 0(0)	13(81.2) 3(18.8)
**Are any particular antiretroviral better than** ** others are for breastfeeding mother, n (%)** Yes No Not sure	4(33.3) 7(58.3) 1(8.3)	8(50.0) 4(25.0) 4(25.0)
**Breastfeeding provides effective** ** contraception, n (%)** Yes No Not sure	10(83.3) 2(16.7) 0(0)	9(56.2) 6(237.5) 1(6.3)

About 15% of the VHTs did not think that a baby could get HIV through breastfeeding and slightly above half (53.8%) considered exclusive breastfeeding as the recommended feeding option for a baby below six months, born to an HIV positive mother. Lastly, majority (58.3%) of the VHTs did not think that any particular antiretroviral regimen was better than others for breastfeeding mothers while 83.3% regarded breastfeeding as an effective contraception method.

**Figure 2.**
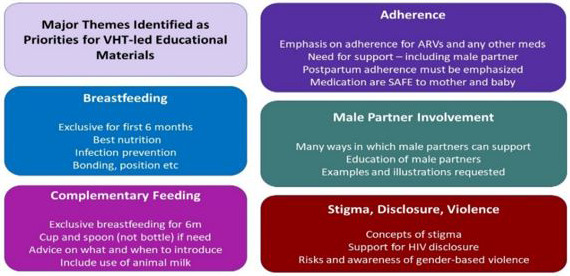
Major Educational Themes. Following initial VHT consultation, the listed five major themes were identified for development of resources.

Following feedback from the VHTs’ initial exercise, we evaluated the information needs of the community members, and the preferred modalities of transmitting that information. We then set out to design tools containing health messages in form of posters, leaflets, and flipcharts with focus on the key topics suggested by the community members; these ranged from the use of photos to illustrate food groups, gender-based violence, male engagement, stigma and discrimination among other topics, as indicated in Figure 2.

### Phase 2

After the refinement of the materials in Phase 1, we returned to Hoima district and presented the revised materials in draft for a further cycle of feedback from the VHTs. VHTs suggested that they preferred their tools to be designed as flipcharts and also suggested that the project provides some incentives to the households visited. Final amendments were then made to the health education tools.

**Figure 3.**
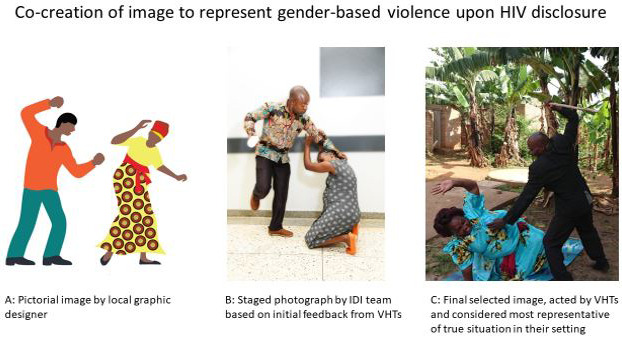
Illustration of the process of co-creating images used in the job-aids.

The project team then returned to Hoima district with the final copies of the materials, including graphic designs by @mcgrathcreate (a young artist based in Cape Town) and in-house photography. Interestingly, although the new materials were well received in general, the request for photographs of ‘people who look like us’ did not seem to have reached the desired audience. Suggestions were made as to how the photographs could be made even more relevant to the communities of Hoima District and the VHTs acted out each of the educational scenarios whilst new photographs were obtained, as illustrated in Figure 3. This was a richly rewarding exercise as it also enabled communication of the extent of the challenges faced in this community, particularly with regard to HIV disclosure to the spouse should a woman be diagnosed with HIV in pregnancy.

After production of the finalised co-created materials – primarily job aids ^23^ and posters ^24, 25^ - we held a further educational workshop and two focus groups (male and female) to gauge final views by the VHTs. We also re-evaluated their knowledge of breastfeeding particularly in the context of HIV at the end of the project. The materials were then given to the VHTs for distribution at the different local health facilities, community centres, police stations as well as in the house holds they previously visited.

***VHTs’ knowledge assessment at project end.*** Generally, there seemed to be an observed increase in the VHTs knowledge on breastfeeding in HIV. All the VHTs were very confident (56.2%) or confident (43.8%) in providing information about breastfeeding in HIV. This shows an improvement from the baseline where about 30% of the VHTs were uncertain of their confidence, as illustrated in Table 2. All the VHTs reported awareness of the recommended feeding option to babies born to HIV positive mothers as well as the ability of a baby getting HIV through breastfeeding.

In contrast, some of the VHTs didn’t believe the father had a role to play in breastfeeding the baby (18.8%). Similarly, half of the VHTs were still not sure if some ARV regimens were better than others for breastfeeding mothers.

### Focus group discussions (FGDs)

We conducted two focus group discussions ^26^ to determine the VHTs’ views on how beneficial the project was to them and their communities. The first FGD was attended by 12 female VHTs while the second was attended by six male VHTs. We structured our results in five domains, namely, previous involvement in VHT routine health education activities, recommended best practices, challenges faced, training needs as well as general support needed.

***Previous involvement in VHT routine health education activities.*** Some VHTs reported to be routinely engaged in various community activities including conducting home visits for pregnant women in the community and encouraging them to attend their antenatal visits, sensitising households on the importance of good nutrition with emphasis on backyard gardening to boost their vegetable and fruits consumption, ensuring children are immunized according to schedule as well as identifying and reporting to authorities in case of any new disease outbreak in their community. Activities specific to breastfeeding included; educating women about the benefits of breastfeeding their babies, encouraging mothers living with HIV to adhere well to their anti-retroviral drugs, as well as supporting mothers to attend postnatal care. Some participants also said that they were previously involved in sensitising community about family planning education, encouraged men to accompany their wives to the hospital during antenatal and postnatal care as well as sensitised men about dangers of gender-based violence.

*“I have been conducting family planning education and encouraging men to accompany their wives to the hospital during antenatal and postnatal care. I also used information in job aids that were provided to sensitize men about the dangers of gender-based violence and I am proud to say that there has been noted a reduction in domestic violence in the community”* – (VHT 05, male)

***Recommended best practices.*** The VHTs appreciated the role of information, education and communication (IEC) materials on breastfeeding in enhancing their engagements with community members during their home visits. The IEC materials on breastfeeding provided by the project, in addition to those on other topics like tuberculosis, COVID-19, and immunisation provided by other partners greatly facilitated the VHTs community education and sensitisation sessions.

*“After the training, I gained knowledge and skills to sensitize mothers and other people in the community through door-to-door home visitation about the importance of a balanced diet, positive living, breastfeeding, and infant feeding. I was also guided by the information education and communication materials that were provided by IDI and the messages are specific for breastfeeding”* – (VHT 03, female)

Incentives like soap and sugar that were provided to the households during the home visits strengthened the communities’ relationships with the VHTs thus making their work easier. Similarly, the logistics, for example job aids, books and pens, provided by the project to the VHTs increased their efficiency during their community activities.

***Challenges faced.*** The VHTs met some challenges during the implementation of their duties. These included; unconducive weather conditions like heavy rains that made it difficult for them to move about their duties, transport challenges as they had to walk long distances to access some homesteads, poor facilitation from the public sector, lack of branding and recognition as an essential component of the health sector, poor living conditions among the homesteads they visited, lack of male involvement in supporting breastfeeding mothers as well as stigma and non-disclosure of HIV among partners thus resulting in GBV.

***Training needs.*** VHTs highlighted the need for further training in topics like nutrition, HIV guidance and counselling, ART use in pregnant and lactating mothers, nutrition as well as communication and public speaking skills.

***General support needed.*** VHTs mentioned the kind of support they desired to conduct their duties and this included provision of IEC materials to facilitate their health education sessions, provision of bicycles to ease transportation to far places, provision of identity cards to act as a form of identification for them, increasing allowances and provision of incentives to motivate them.

### Durability of the intervention

Eight months after formal project close out, in February 2023, participating VHTs were invited for an informal focus group to review the impact of the project and discuss future priorities in this population. VHTs reported that they continued to benefit from using the job aids and posters. They described increased confidence in provision of structured information since the trainings conducted by the project, as illustrated in some of the quotes below;

*"The job aids continue to be an ideal tool in my community work. They help guide me to review, refine, and reinforce the various topics that I share with various community members. They reduce the chances of even me as a VHT making an error as I easily refer back to them to ensure that I am sharing the right information." – (VHT 11, female).*
*"The job aids have made my training easier for people to understand. They have well written text, pictures, and diagrams that are easy to explain and understand. Even when the community members have more inquiries, it is easier for me to share with them the right information as and when they need it." – (VHT 01, female).*
*"The job aids that were provided by IDI help create greater clarity when I am sensitizing community members. They provide detailed messages that make it quicker for the community members that I discuss with to learn and recall the information that I share with them." – (VHT 06, male).*

All reiterated that they would be keen to participate in future co-creation and participatory research projects within their communities.

## Discussion

The aim of this project was to use the local expertise and networks of VHTs to determine priorities and concerns regarding breastfeeding among women living with HIV at community level, with the help of co-created resources, to communicate about the priority health topics, including medication use during breastfeeding. Involvement of communities in co-creation of resources is encouraged as it is one of the drivers for innovation and acceptability of these resources in the targeted communities ^3^. Our project involved VHTs in the designing of resources used for health education in their communities and through this, many innovative ideas were birthed, for example, use of locally-generated pictorial content to illustrate various activities like GBV and male involvement in breastfeeding.

There was an observed increase in the knowledge on breastfeeding in HIV among the VHTs engaged in the study. This could be attributed to the trainings provided to the VHTs as well as the job aids designed with information that were given to each of the VHTs during the project period. This was similarly observed in another study conducted in south eastern Uganda where VHTs had good knowledge on chronic diseases among older people, following effective training on this topic ^27^.

VHTs engaged in our project expressed the need for incentives like transport allowances, notebooks, pens, gumboots and raincoats to help them effectively carry out their duties. This was similarly observed in another study conducted among VHTs in Mukono and Wakiso districts in Uganda ^28^. Taking on VHT roles is considered a voluntary assignment, thus VHTs are expected to work with minimal pay, this has a negative effect on their efficiency given the various challenges they are met with during their engagements ^29^. Provision of incentives is instrumental in motivating VHTs to take on their activities. A study conducted in Tororo district, Uganda showed a positive relationship between incentives (both monetary and non-monetary) and VHTs’ performance in promotion of health services ^30^.

### Lessons learnt

We were open to developing resources that could be accessed *via* a smartphone, for example, but this initial exercise showed that verbal and paper-based materials remained the most accessible options in this population.

Secondly, VHTs, if equipped and trained adequately, are a very effective strategy in disseminating health education to the communities, especially in rural settings as they are easily trusted by rural communities hence provide a link between healthcare workers and the community.

Finally, VHTs are deeply aware of the challenges impacting their communities and of how IEC materials are received. Co-creation is a powerful tool to ensure that the information is presented in the best way to reach the desired community.

## Conclusion

Training and engaging community health workers is an effective strategy in disseminating important health information to communities, as shown in a rural Ugandan setting. Involving the communities in designing programs targeted for them is an effective strategy to ensure clear, targeted information is delivered in an accessible format for the target population.

This pilot will shape future public engagement and involvement work in rural settings in Uganda and Africa at large.

## Data Availability

Our data is restricted due to institutional data sharing limits, however, it can be available on request by emailing enalugga@idi.co.ug, subject to agreement to the IDI data sharing terms. Figshare: **Focused Group Discussion guide.docx.** https://doi.org/10.6084/m9.figshare.23931972.v2 This project contains the following extended data: Data file 1: Focused Group Discussion guide.docx Figshare: **VHTs knowledge assessment questionnaire.docx.** https://doi.org/10.6084/m9.figshare.23931966.v2 This project contains the following extended data: Data file 3: VHTs knowledge assessment questionnaire.docx Figshare: **Male engagement poster in English.pdf.** https://doi.org/10.6084/m9.figshare.23931888.v2 This project contains the following extended data: Data file 4: Male engagement poster in English.pdf Figshare: **Breast feeding an infant poster in Runyoro.pdf**. https://doi.org/10.6084/m9.figshare.23931867.v2 This project contains the following extended data: Data file 5: Breast feeding an infant poster in Runyoro.pdf Figshare: **VHTs Job aid English Version.pdf.** https://doi.org/10.6084/m9.figshare.23926209.v2 This project contains the following extended data: Data file 6: VHTs Job aid English Version.pdf Data are available under the terms of the Creative Commons Zero “No rights reserved” data waiver (CC0 1.0 Public domain dedication).
