# Supplementary material for: It Takes A Village; Involvement of Village Health Teams to Develop Tools and Resources to Communicate about Antiretroviral Use in Pregnancy and Breastfeeding at Community Level in Uganda
Source: Wellcome Open Res. 2023 Nov 9;8:510. [Version 1] doi: 10.12688/wellcomeopenres.19088.1 (PMC10825416; doi:10.12688/wellcomeopenres.19088.1)
Supplement: Peer review history

# Reviewer report: Reviewer response for version 1

**DOI:** 10.21956/wellcomeopenres.21163.r71753

**Published:** 2024-01-29

**Authors:** Alinda Young

**Affiliations:** 1 RTI International, Research Triangle Park, North Carolina, USA

Around half of women worldwide may need medication during breastfeeding, and in Uganda, where HIV prevalence in women of childbearing age was 7.1% in 2020-2021, the risk of infection is high among adolescent girls and young women. The lowered immunity during pregnancy makes women susceptible to infections like tuberculosis and malaria, leading to a dilemma for breastfeeding mothers as many medications lack clear data on their safety for infants, causing anxiety and potentially resulting in untreated diseases. The World Health Organization recommends strategies such as home visits by Village Health Teams (VHTs) to convey crucial health messages, enhancing maternal and child health. VHTs serve as a trusted bridge between healthcare workers and rural communities. However, sustainable, and affective dissemination requires a co-creation approach between the scientist and the community. Effectively training and involving community health workers in program design in rural Uganda proved to be a successful strategy for disseminating clear and targeted health information to the communities.

Is the work clearly and accurately presented and does it cite the current literature?

Yes

If applicable, is the statistical analysis and its interpretation appropriate?

Yes

Are all the source data underlying the results available to ensure full reproducibility?

Yes

Is the study design appropriate and is the work technically sound?

Yes

Are the conclusions drawn adequately supported by the results?

Yes

Are sufficient details of methods and analysis provided to allow replication by others?

Yes

Reviewer Expertise:

Sexual and Reproductive Health, Maternal and Child Health, HIV, HIV Prevention Methods

I confirm that I have read this submission and believe that I have an appropriate level of expertise to confirm that it is of an acceptable scientific standard.

# Reviewer report: Reviewer response for version 1

**DOI:** 10.21956/wellcomeopenres.21163.r71750

**Published:** 2024-01-11

**Authors:** Françoise Renaud

**Affiliations:** 1 World Health Organization, Geneva, Switzerland

In low-resource settings, young mothers may be particularly vulnerable to the lack of health education and access to recent research findings for making good decisions, notably on the safety of lifesaving ARV medication use during breastfeeding for women living with HIV. From 1999 in Uganda, building on informal networks of trusted community members, Village Health Teams (VHTs) became integral parts of the national health policy strategy for implementing primary health care. The authors co-created communication materials with a team of VHTs (Hoima, Uganda) on novel data on medication use in breastfeeding (generated from their research projects) for use in their house-to-house visitation and health education. Initial training workshop and review of draft materials with VHT pairs, home visits by  VHT pairs for feedback from the target population, and redesign of materials were used. The authors were able to identify clear and understandable messaging, better understand health priorities, and the need for well-designed participatory research and communication strategy of research findings to the population who will benefit from them

 The method section clearly describes the different steps from ethical consent, choice of the district, selection of VHTs, and co-creation activities with pre- and post-piloting of materials, that lead to the completion, translation, and printing of the final materials, followed by focus group discussions to understand how beneficial the project has been to VHTs. In the result section, major key findings are well structured and analyzed.  Attention was given to the values and preferences of the targeted population, such as verbal and visual help, the request for photographs of “who look like us”, or various sensitive issues including stigma, HIV disclosure to the spouse should a woman be diagnosed with HIV during pregnancy, male involvement in breastfeeding, or gender-based violence. VHTs’ knowledge assessment at the project end showed an increase in their confidence to provide information on HIV and breastfeeding and awareness of the recommended feeding option for babies.

 Focus Group Discussions with VHTs highlighted how incentives to the household (sugar, soap) could make their job easier, and logistics to the VHTs (books, pens, IEC materials) facilitated their engagement with communities and efficiency. More logistics (bicycles, raincoats, ID cards), training in specific topics (nutrition), or communication skills are needed to address major challenges met (transport challenges, lack of recognition, poor social conditions of the visited home. The authors conclude that community health workers are an effective strategy for disseminating important health information.

 Question to the authors: the discussion section states that there was an observed increase in the knowledge on breastfeeding in HIV among the VHTs engaged in the study.  Could you further elaborate on the impact of the co-created activities and project on the population benefiting from the activities, the end-user? Alternatively, provide information on the plans to evaluate the impact on treatment uptake or adherence and health outcomes among young mothers living with HIV and their breastfed babies.  More research on impact seems needed that could be included in the conclusion.

Is the work clearly and accurately presented and does it cite the current literature?

Yes

If applicable, is the statistical analysis and its interpretation appropriate?

Yes

Are all the source data underlying the results available to ensure full reproducibility?

Yes

Is the study design appropriate and is the work technically sound?

Yes

Are the conclusions drawn adequately supported by the results?

Yes

Are sufficient details of methods and analysis provided to allow replication by others?

Yes

Reviewer Expertise:

Public health, HIV, leading WHO Pregnancy and Therapeutic Working Group

I confirm that I have read this submission and believe that I have an appropriate level of expertise to confirm that it is of an acceptable scientific standard.
